# Ferrocenophanium Stability and Catalysis

**DOI:** 10.3390/molecules28062729

**Published:** 2023-03-17

**Authors:** Sai Anvesh Bezawada, Neira Ušto, Chloe Wilke, Michael Barnes-Flaspoler, Rajamoni Jagan, Eike B. Bauer

**Affiliations:** Department of Chemistry and Biochemistry, University of Missouri, One University Boulevard, St. Louis, MO 63121, USA

**Keywords:** ferrocenophanium cations, catalyst stability, propargylic substitution reactions

## Abstract

Ferrocenium catalysis is a vibrant research area, and an increasing number of ferrocenium-catalyzed processes have been reported in the recent years. However, the ferrocenium cation is not very stable in solution, which may potentially hamper catalytic applications. In an effort to stabilize ferrocenium-type architectures by inserting a bridge between the cyclopentadienyl rings, we investigated two ferrocenophanium (or *ansa*-ferrocenium) cations with respect to their stability and catalytic activity in propargylic substitution reactions. One of the ferrocenophanium complexes was characterized by single crystal X-ray diffraction. Cyclic voltammetry experiments of the ferrocenophane parent compounds were performed in the absence and presence of alcohol nucleophiles, and the stability of the cations in solution was judged based on the reversibility of the electron transfer. The experiments revealed a moderate stabilizing effect of the bridge, albeit the effect is not very pronounced or straightforward. Catalytic propargylic substitution test reactions revealed decreased activity of the ferrocenophanium cations compared to the ferrocenium cation. It appears that the somewhat stabilized ferrocenophanium cations show decreased catalytic activity.

## 1. Introduction

In recent years, there has been an increasing interest in catalytic processes based on 3d transition metals, which are abundant and relatively inexpensive [1]. Consequently, like with other 3d transition metals, iron catalysis has emerged as a vibrant research area in the past decades [2,3,4,5,6,7,8,9,10,11,12,13,14,15,16,17]. Iron-based catalysis has a number of advantages compared to other transition metals typically utilized in catalysis. It is relatively non-toxic, abundant, inexpensive, and environmentally benign. Accordingly, several organic transformations have been reported to be catalyzed by iron-based catalyst systems, such as oxidations, reductions, substitutions, cross-coupling, or polymerization reactions. Iron-based catalysts can range from simple salts like FeX_2_ or FeX_3_ [18,19,20], Fe(OTf)_2_ [21,22], Fe(OAc)_2_ [23,24,25,26], or Fe(acac)_3_ (acac = acetylacetonato) [27] to higher-sophisticated metal complexes such as Knölker’s or related complexes [28,29], iron complexes derived from phosphorus- [7,30] or nitrogen-coordinating ligands [31,32] or iron coordination compounds which can catalyze reactions site- or substrate-selectively [33,34,35] and enantioselectively [36,37,38].

We have employed a number of iron-based catalysts in the past in oxidation reactions [39,40,41,42,43,44,45], and during the course of our studies, we found that ferrocenium hexafluorophosphate (FcPF_6_) catalyzes propargylic substitution reactions [46,47]. Ferrocenium cations have been increasingly applied as catalysts in a number of other reactions [48], such as Friedel-Crafts [49,50] and allylic alkylations [51], amine [52] or CH oxidations [53], cyanosilylation of carbonyl compounds [54], reductive etherification [55], aminolysis [56] or ring opening of epoxides [57], aromatic iodinations [58], the Strecker reaction [59], the Mannich reaction [60], photodecomposition [61], ring expansion [62], or polymerization reactions [63,64,65,66]. Antiproliferative effects of certain ferrocenophanes have been reported [67]. The tunability of the ferrocenium platform provides an advantage when it comes to catalyst improvement.

However, ferrocenium cations are not very stable in solution, especially in chlorinated solvents and under aerobic conditions [68,69,70,71,72,73]. The ferrocenium cation decomposes in solution, and this fact has also been taken advantage of in Diels-Alder reactions [73]. Catalyst decomposition is a big concern, as it may counter efforts to design catalysts for specific purposes. As such, efforts to investigate catalyst decomposition and the design of more robust catalyst systems are important issues.

**Scheme 1:**
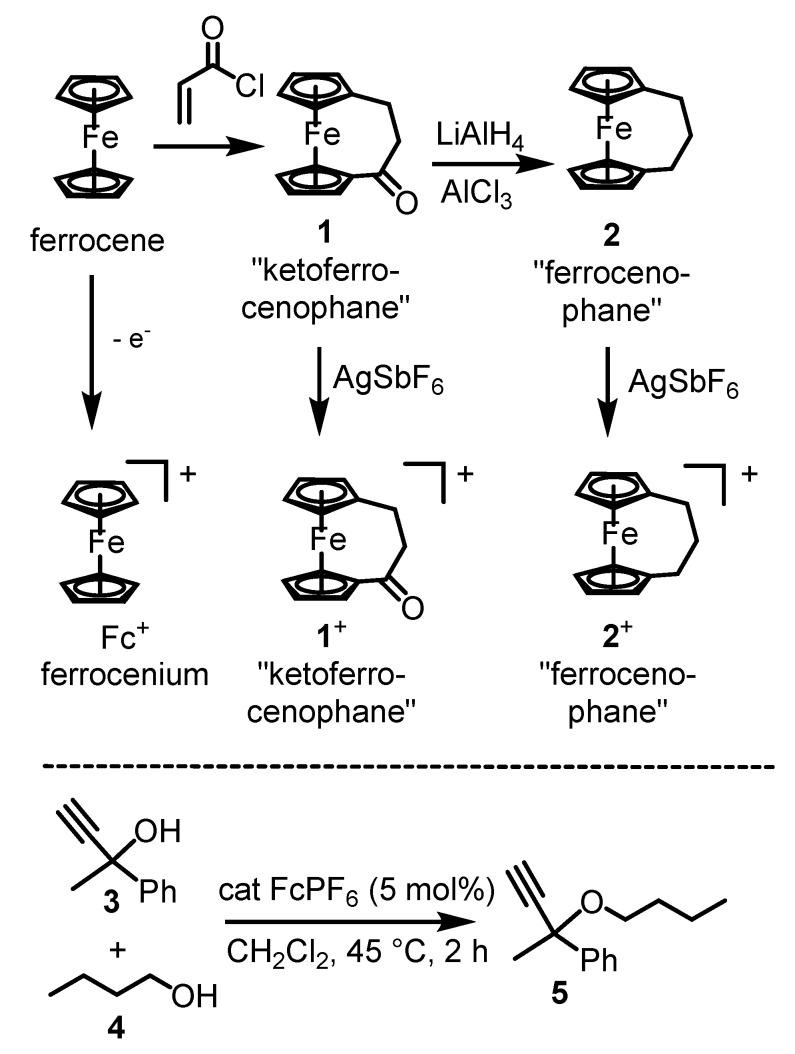
Ferrocene and ferrocenophanium complexes and catalytic application.

Herein, we investigate ferrocenophanium cations as potentially more stable complexes compared to ferrocenium and test them as catalysts in propargylic substitution reactions. Ferrocenophanes (or *ansa*-ferrocenes) are ferrocene complexes, where the two cyclopentadienyl (Cp) ring systems are bridged (e.g., **1** and **2** in Scheme 1, top). We hypothesized that a bridge between the Cp rings stabilizes the complex, and would slow down or block decomposition pathways, such as decomposition by Cp ring loss or intermolecular decomposition by nucleophilic attack on the iron (which would in a ferrocenophane be somewhat protected by the bridge). We determined the stability of ferrocenophanium cations in the absence and presence of nucleophiles by cyclic voltammetry, and we applied the cations in propargylic substitution test reactions (Scheme 1, bottom).

## 2. Results and Discussion

Ferrocenophanes or *ansa*-ferrocenes are a well-known compound class [74]. The bridges can be all-carbon [74,75] or contain heteroatoms [76,77], and several synthetic methods for their access have been described [78]. Either the two ring systems are connected by a series of organic transformations, or a preformed, bidentate dicyclopentadienyl ligand is coordinated to an iron center (“flytrap method”) [79]. We set out to synthesize and investigate the known, mono-bridged “ketoferrocenophane” complex **1**, which can be obtained from ferrocene in an acylation-Michael reaction sequence (Scheme 1) [80,81]. The keto functionality can be reduced to obtain the hydrocarbon-only bridge in the “ferrocenophane” **2** (Scheme 1) [80,81]. The chemistry to obtain ferrocenophanes **1** and **2** worked as described in the literature. The conversion from **1** to **2** can be followed by IR, as the C=O stretch in **1** disappears after reduction. However, the workup procedures are tedious, and the yields are generally not very high, as also indicated in the original synthetic procedures. Oxidation of the two complexes led to the corresponding ferrocenophanium salts **1**^+^SbF_6_ [82] and **2**^+^SbF_6_ [83,84]. The oxidation of **1** and **2** can be observed by UV-vis spectroscopy, as **1**^+^SbF_6_ and **2**^+^SbF_6_ give a new band around 620 nm, as it is characteristic of ferrocenophanium cations [83]. NMR investigation of the ferrocenophanium salts is, due to their paramagnetic nature, not possible. Consequently, our investigations were plagued by difficulties accessing the ferrocenophanium ions in large quantities.

**Figure 1:**
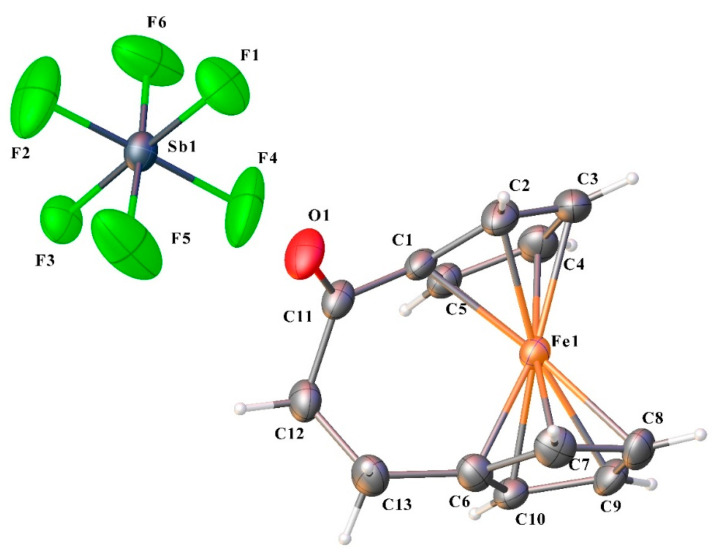
Molecular structure of **1**^+^SbF_6_ showing the displacement ellipsoids drawn at 50% probability level with atom labeling scheme. Key bond lengths (Å) and angles (°): C(1)-Fe(1): 2.033(10), C(6)-Fe(1): 2.026(13), C(11)-O(1):1.219(13), Cp(centroid)-Fe: 1.693; Cp-Cp angle: 14.39(6), C11-C12-C13: 111.0(11).

The molecular structure of **1**^+^SbF_6_^–^ determined by single crystal X-ray diffraction analysis is shown in Figure 1. The details of intensity data collection and refinement are given in the Supporting Information. The crystal structure refinement parameters are summarized in Table 1.

The complex **1**^+^SbF_6_ crystallizes in monoclinic spacegroup P21/c with one ferrocenophanium complex and one SbF_6_^–^ anion in the asymmetric unit. From the crystal structure it is observed that the monosubstituted C_5_H_4_ rings are η^5^ coordinated to the central iron atom with C(Cp)-Fe bond lengths ranging from 2.020(6) Å to 2.127(8) Å, showing a significant range. The short C(Cp)-Fe bond length values such as C1(Cp)-Fe1 = 2.020(6) Å and C6(Cp)-Fe1 = 2.040(9) Å is attributed to the substitutions at C1 and C6 positions, respectively. The metallocene conformation is eclipsed with a slightly distorted ferrocene framework showing a Cp(centroid)-Fe-Cp(centroid) angle of 171.8(2)˚ and Cp(centroid)-Fe distance of 1.692(5) Å and 1.6939(13) Å respectively. The carbonyl group is trigonally planar with the C1-C11-C12 [117.4(6)°] angle being slightly smaller than C1-C11-O1 [121.2(9)°] and O1-C11-C12 [120.9(9)°], respectively. Interestingly, the trigonal planar carbonyl group is oriented out of the adjacent Cp-planes with calculated dihedral angles of 41.56(3)° and 51.44(3)°, respectively. In the SbF_6_^–^ anions, the bond lengths and angles are within the expected range with Sb-F distances ranging from 1.771(11) Å to 1.878(5) Å. In the three-dimensional crystal structure, the molecules are mainly stabilized by C-H…O and C-H…F interactions (Supporting Information Table S1). The oxygen atom O1 acts as a bifurcated acceptor forming two C-H…O interactions such as C10-H10…O1 and C12-H12A…O1 which connects the adjacent ferrocenophanium complexes (Supplementary Materials, Figure S1). Adjacent ferrocenophanium chains are further bridged by SbF_6_^–^ anions via three C-H…F interactions (C3-H3…F3, C5-H5…F1, and C7-H7…F3), which results in the formation of a three-dimensional network in the crystalline solid. The interlinkage of the ferrocenophanium complex chain and the SbF_6_^–^ anions via C-H…F interaction is illustrated in Figure S2.

**Figure 2:**
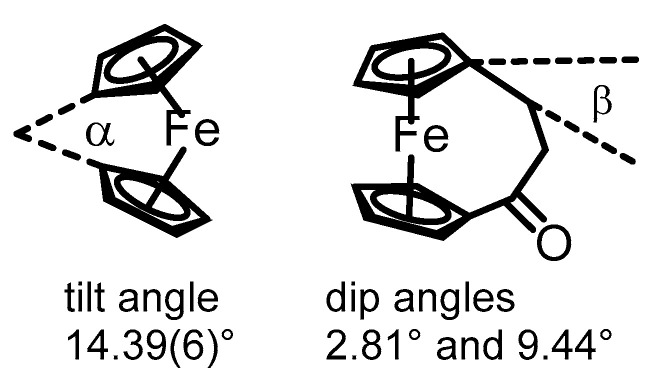
Geometric parameters.

The bridge in the ferrocenophanium cation **1**^+^ causes the Cp rings to be inclined to each other with a dihedral (or tilt) angle of 14.39(6)° (α in Figure 2) [85,86]. Therefore, there is some open space on the opposite side of the bridge, where we hypothesized catalysis can take place. The tilt is smaller compared to ferrocenophanes with bridges consisting of two atoms, where angles around 22° have been reported [87]. Ferrocenophanes with a bridge consisting of only one atom exhibit angles between 27 and 38°, putting a substantial strain on the system [79,88]. Obviously, the shorter the bridge, the larger the tilt. In general, atoms directly bonded to the Cp ligands are located in the plane of the ring system. In ferrocenophanes, these atoms can be bent out of the plane of the Cp ring system and quantified with the dip angle [89]. The dip angle is defined as the deviation of the angle between the centroid of the Cp ring, the ipso-carbon atom, and the first carbon atom of the bridge from the ideal 180° (β in Figure 2) [86,89]. The angles were calculated to be 2.81° (C13) and 9.44° (C11, the C=O carbon). It appears that the C1 carbon of the Cp ring bearing the C=O unit has a slightly higher sp^3^ character.

### 2.1. Cyclic Voltammetry Experiments

We next investigated the stability of the ferrocenophanium cations by cyclic voltammetry (CV) [90,91]. Related experiments have been performed with other ferrocenophanes [92,93]. In a typical CV experiment, the ferrocenophanes are oxidized to the corresponding ferrocenophanium cations. The reversibility of this process, as indicated by the ratio of the *i*_c_/*i*_a_ current, would give insight into the stability of **1**^+^ and **2**^+^, compared to the ferrocenium cation. A fully reversible process would give an *i*_c_/*i*_a_ ratio of 1. As shown in Scheme 1 (bottom), the catalytic propargylic substitution test reaction involves the propargylic alcohol **3** and *n*-butanol (**4**) as the nucleophile. We also investigated the stability of **1**^+^ and **2**^+^ in the presence of alcohol nucleophiles to investigate whether they are part of potential decomposition chemistry. An *i*_c_/*i*_a_ ratio smaller than 1 may indicate an electrochemical oxidation followed by an irreversible chemical reaction (EC mechanism) [91].

**Figure 3:**
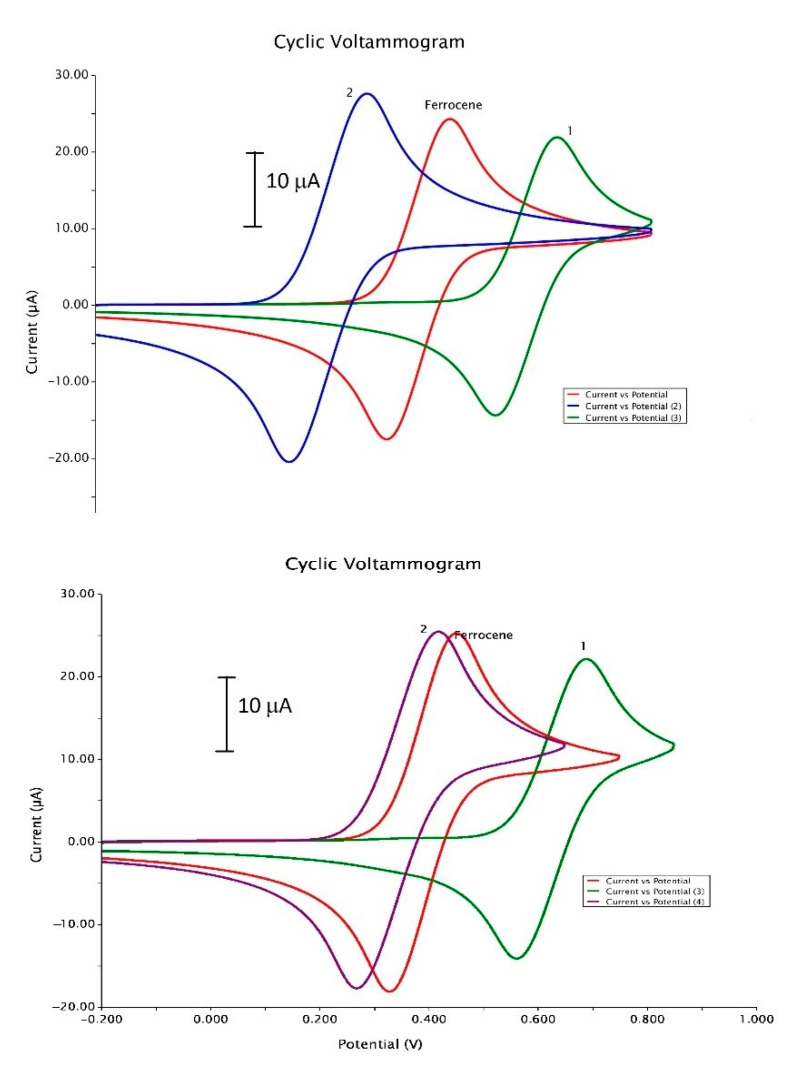
Cyclic voltammograms (scan rate: 100 mV s^–1^) in CH_2_Cl_2_ solutions (1.0 mM) at 25 °C (supporting electrolyte *n*-Bu_4_NPF_6_, 0.1 M), not calibrated. (**Top**): traces from the left **2**, ferrocene, and **1**. (**Bottom**): from the left **2**, ferrocene, and **1** in the presence of *n*-BuOH.

Representative CV traces of ferrocene, **1** and **2** in CH_2_Cl_2_ are shown in Figure 3, in the absence (top) and presence of *n*-BuOH (bottom). As can be seen, all three compounds give the typical “duck-shaped” voltammograms expected for diffusion-controlled, reversible electron-transfer processes [91]. Plots of the peak current *i*_p_ versus the square root of the scan rate give linear graphs for diffusion-controlled redox processes [91], which we observed (representative graphs are given in the Supporting Information). The numerical results of the CV experiments are compiled in Table 2 and Table 3.

In Table 2, CV data for ferrocene, and the ferrocenophane complexes **1** and **2** are compiled. This data allows for the comparison of the stability of the ferrocenium cation Fc^+^ and the ferrocenophanium complexes **1**^+^ and **2**^+^ in solution in the absence of any other substrate. The ΔE values are a little higher than expected from theory for a reversible electron transfer (59 mV), which may be caused by the poorer conductivity of CH_2_Cl_2_ and CH_3_CN solutions of *n*-Bu_4_NPF_6_ utilized as the electrolyte; it may also indicate slow electrode kinetics [91]. The ΔE values are somewhat larger in CH_2_Cl_2_ compared to CH_3_CN, which we ascribe to the lower conductivity of CH_2_Cl_2_, which leads to higher ohmic resistance of the solutions [91]. The complexes are barely soluble in other solvents.

The *E*_1/2_ values roughly correlate with the oxidation potential of metal complexes. For ferrocene, the standard redox potential is defined as +0.46 V in CH_2_Cl_2_ and +0.40 V in CH_3_CN with *n*-Bu_4_NPF_6_ as the electrolyte [90]. As derived from the *E*_1/2_ values, the ketoferrocenophane **1** as a higher oxidation potential of 0.604 V and 0.658 V in CH_2_Cl_2_ and CH_3_CN, respectively (Table 2, entries 2 and 5). These values are higher than that for ferrocene itself, which may be caused by the keto functionality in **1**; it may withdraw electron density from the Cp ring system connected to it, resulting in a higher oxidation potential of the ketoferrocenophane complex **1**. In turn, for ferrocenophane **2**, the redox potentials in CH_2_Cl_2_ and CH_3_CN (+0.318 V and +0.395 V, respectively; entries 3 and 6) are lower than for ferrocene. Here, the electron-donating alkyl bridge may overall increase the electron density of the Cp ring systems, resulting in a lower oxidation potential.

The *i*_c_/*i*_a_ ratios allow for the assessment of the stability of the complexes in the solution. If the *i*_c_/*i*_a_ ratio is smaller than 1, decomposition of the oxidized species may occur, so that there is less material left after oxidation to be reduced back to the starting material, resulting in a lower reduction current, following an EC mechanism described above [91].

As can be seen, the *i*_c_/*i*_a_ ratios for the three complexes are fairly similar, ranging from 0.98 to 0.86 in any solvent. It seems that for the typical timeframe of the CV experiment (several seconds to minutes, depending on the sweep rate), only a small fraction of the oxidized ferrocene, ketoferrocenophanium **1**^+^, and ferrocenophanium **2**^+^ decompose under the reaction conditions. This behavior may not be unexpected. Ferrocene is known to be fairly stable under CV conditions with fast sweep rates, and it appears that the establishment of a bridge between the Cp rings does not change that significantly during the short time frame of a CV experiment.

However, we were interested to determine how the stability changes in the presence of the catalysis substrates. As mentioned, we applied the ferrocenium cation as a catalyst for propargylic substitution reactions (Scheme 1, bottom), where, besides the catalyst, the tertiary propargylic alcohol **3** and alcohol nucleophiles such as *n*-butanol (**4**), MeOH or *i*-PrOH were present [46,47]. Related CV experiments have been performed by others with ferrocenophanes in the presence of an imidazole base [94]. We performed the same CV experiments as in Table 2 in the presence of propargylic alcohol **3** and a number of primary and secondary aliphatic alcohols. We were interested in whether the stability of the ferrocenophanium complexes changes in the presence of alcohols, as reflected by the *i*_c_/*i*_a_ ratios. The results are compiled in Table 3, representative CV traces are shown in Figure 3 (bottom) in the presence of *n*-BuOH. As can be seen, the duck shape is, in general, maintained in the presence of *n*-BuOH.

For ferrocene, the ΔE and the *E*_1/2_ values are comparable to the measurements in the absence of alcohol. In the presence of MeOH, *i*-PrOH, and the propargylic alcohol **3**, in CH_2_Cl_2_ or CH_3_CN, the ferrocene seems to be fairly stable, as judged by the *i*_c_/*i*_a_ ratios, which range from 0.78 to 0.96. In CH_3_CN as the solvent, there is a notable difference for *n*-BuOH as the nucleophile. In the presence of *n*-butanol, the *i*_c_/*i*_a_ ratio dropped to 0.78 (entry 2). Interestingly, in the presence of MeOH, the *i*_c_/*i*_a_ ratio stayed at around 0.94 (entry 1). The drop in stability in *n*-BuOH can be rationalized by a nucleophilic attack of the alcohol on the ferrocenium cation. It appears that MeOH would not undergo such an attack, which may be somewhat counterintuitive. We speculated that MeOH nucleophile is better solvated by the CH_3_CN solvent, though. In turn, in the presence of *i*-PrOH, the stability of the ferrocenium cation is also high, as reflected by an *i*_c_/*i*_a_ ratio of 0.95 to 0.96 (entries 3 and 7). This may be a consequence of the low nucleophilicity of the secondary *i*-PrOH compared to the primary alcohol *n*-butanol.

Interestingly, the ketoferrocenophanium cation **1**^+^ shows in CH_3_CN a lower, but consistent stability, as demonstrated by *i*_c_/*i*_a_ ratios around 0.81 (entries 8 to 12). The ratios stay constant across different nucleophiles. Presumably, a chemical change of **1^+^** takes place without the nucleophile being directly involved. The stability for different alcohol nucleophiles is slightly higher in CH_2_Cl_2_ (*i*_c_/*i*_a_ ratios of 0.75 to 0.87 entries 13 to 16); here, the presence of the propargylic alcohol **3** leads to a slight decrease in stability, for which we do not have a clear explanation. Lower solvation of **3** may play a role here again.

However, the cation derived from the ferrocenophane **2** seems to be the most stable under these conditions, with average *i*_c_/*i*_a_ ratios of 0.91 to 0.97 in CH_3_CN and CH_2_Cl_2_ in the presence of *n*-BuOH and MeOH. It appears that the ferrocenophanium cation **2**^+^ is somewhat more stable than the ferrocenophanium cation **1**^+^ and the ferrocenium cation itself in the presence of MeOH and *n*-BuOH.

A possible explanation may be that the alkyl bridge in **2** protects the complex from bimolecular decomposition, assuming that the decomposition process involves the attack of *n*-BuOH on the iron center. The slightly more extended decomposition of the ferrocenium cation in the presence of *n*-BuOH may be due to the lack of the protecting bridge, facilitating the attack on the iron center. The ketoferrocenophanium complex **1**^+^ seems overall to be somewhat less stable than the ferrocenophanium cation **2**^+^. Here, the electron-withdrawing keto group on the Cp ring may make the iron center more electrophilic, facilitating an attack of the alcohol on the iron center, despite some protection from the bridge. Also, involvement of the carbonyl group in the decomposition process, for example, through the formation of a hemiacetal, may play a role.

We also performed the experiments in Table 3 at different sweep rates, and for ferrocene and ferrocenophane **1** in presence of *n*-BuOH, the results are compiled in Table 4. Here, for ferrocene, a clear dependency of the sweep rate on the *i*_c_/*i*_a_ ratios can be observed in CH_3_CN. The *i*_c_/*i*_a_ ratios increase with increasing sweep rate. At a slower sweep rate, the ferrocenium ion is exposed for a longer time to the *n*-BuOH nucleophile, resulting in decomposition to a larger extent, which leads to lower *i*_c_/*i*_a_ ratios. The *i*_c_/*i*_a_ ratios for the ferrocenophanes **1** and **2** are relatively independent of the sweep rate with the exception of ketoferrocenophane **1**, where the *i*_c_/*i*_a_ ratio decreases with increasing sweep rate in CH_2_Cl_2_. It may be speculated that for **1**^+^ and **2**^+^, decomposition by a nucleophilic attack is, within the timeframe of the CV experiment, diminished due to the protecting bridge in the ferrocenophanes.

The results in Table 2, Table 3 and Table 4 are of interest in several respects. The bridges in the ferrocenophanes provide some protection for their respective cations in solution, and the protecting effect seems to be dependent on the solvent and the type of the nucleophile. However, the trend is less pronounced than expected. Ferrocene seems to be slightly more stable under the conditions in Table 3 than the ketoferrocenophane **1**. It appears that the ferrocenium cation decomposes faster in the presence of *n*-BuOH, so the bridge in **1** may provide some protection over extended periods of time. In some instances, the decomposition increases with decreasing sweep rate. The findings may have some mechanistic impacts. We assume for the propargylic substitution reaction in Scheme 1 (bottom) an ionic mechanism, where first a propargylic carbocation is generated, which then can be attacked by the alcohol nucleophile [46]. The ferrocenium or ferrocenophanium cations to be applied for chemistry like in Scheme 1 (bottom) may undergo a chemical change substantially in the presence of *n*-butanol prior to or during catalysis.

### 2.2. Catalytic Experiments

In order to find out whether the catalyst stability has an impact on the activity, we employed the **1**^+^SbF_6_ and **2**^+^SbF_6_ in the propargylic test reaction in Scheme 1 (bottom) and two more reactions and compared them to the efficiency of the ferrocenium cation previously established in our research group. The results are compiled in Table 5. The reaction of the propargylic alcohol **3** with *n*-BuOH gave previously the propargylic ether product **5** [47]. The propargylic substitution reaction of the cyclopropyl-substituted propargylic alcohol **6** also resulted previously in the ring-opened eneyne product **7** when ferrocenium hexafluorophosphate (FcPF_6_) was employed as a catalyst; the thiophenyl-substituted propargylic alcohol **8** gave the substitution product **9** with the cyclopropyl ring still intact [46].

Overall, the complexes **1**^+^SbF_6_ and **2**^+^SbF_6_ appear to be not as catalytically active as the ferrocenium cation itself. While some product was observed by GC, we were able to isolate the products in only 1 to 44% yields. The reactions in Table 5 are routine in our laboratory, and when employing FcPF_6_, we obtain frequently yields of at least 30% and higher [46,47]. Of the alcohols employed in Table 5, propargylic alcohol **3** showed the least activity in substitution reactions in our previous experiments [46,47]. Obviously, **1**^+^SbF_6_ and **2**^+^SbF_6_ seem not reactive enough to activate the propargylic alcohol **3** toward substitution. The cyclopropyl-substituted propargylic alcohol **6** is activated toward nucleophilic substitution [46], and both catalysts **1**^+^SbF_6_ and **2**^+^SbF_6_ result in moderate yields for the formation of eneyne **7**. The thiophenyl-substituted propargylic alcohol **8** is also activated toward nucleophilic substitution [46]; however, both catalysts gave no detectable product **9** or only very little of it. The yield of **7** is slightly higher for **2**^+^; however, the general trend points toward a lower catalytic activity.

The low yields in Table 5 for the ferrocenophanium complexes may indicate that the somewhat increased stability of the complexes decreases their activity. Catalysts must exhibit a level of reactivity in order to show catalytic activity. Another point to be considered is the actual catalytically active species when FcPF_6_ is employed as a catalyst. While the ferrocenium cation may show some stability under cyclic voltammetry conditions (room temperature and short reaction time), it may undergo chemical changes under the catalytic conditions in Table 5 (45 °C and at least 2 h reaction time). Longer reaction times with ketoferrocenophanium complex **1**^+^ did not result in higher yields, though. Whether the decomposition products of the ferrocenium or ferrocenophanium cations are the actual catalytically active species is currently under investigation in our research group. If the ferrocenophanium complexes show somewhat increased stability in solution, as hypothesized, the reactivity may just be lower.

## 3. Conclusions

Overall, cyclic voltammetry data presented herein establish a moderate protecting effect of the bridge in ferrocenophanium cations in presence of alcohol nucleophiles compared to ferrocenium. These findings were established by CV *i*_c_/*i*_a_ values and their dependency on the sweep rate. However, catalytic experiments revealed that the ferrocenophanium cations showed overall lower catalytic activity compared to the ferrocenium cation, as judged by lower isolated catalysis product yields. While decreased catalytic stability may result in decreased catalytic activity, very reactive catalysts in solution may also show a higher tendency to decompose. The catalytic activity landscape of ferrocenium and ferrocenophanium cations investigated herein may be more complex than it appears at first glance.

## Figures and Tables

**Table 1:** Crystal Structure Refinement of **1^+^**SbF_6_.

Parameters	Complex 1^+^SbF_6_
Empirical formula	C_13_H_12_F_6_FeOSb
Formula weight	475.83
Temperature (K)	296.15
Wavelength (Å)	1.54178
Crystal system, space group	Monoclinic, P2_1_/c
Unit cell dimensions a (Å)	7.2292(2)
b (Å)	20.6452(5)
c (Å)	10.7792(3)
α (°)	90
β (°)	107.127(2)
γ (°)	90
Volume (Å^3^)	1537.43(7)
Z, Calculated density (Mg/m^3^)	4, 2.056
Absorption coefficient (mm^–1^)	22.107
F(000)	916.0
Crystal size (mm)	0.08 × 0.050 × 0.020
Theta range for data collection (°)	4.883 to 68.48 deg.
Limiting indices	–8 ≤ h ≤ 8, –24 ≤ k ≤ 24, –12 ≤ l ≤ 12
Reflections collected/unique	15701/2821 [R(int) = 0.0815]
Completeness to theta = 68.48 (%)	100.0
Refinement method	Full-matrix least-squares on F^2^
Data/restraints/parameters	2821/0/199
Goodness-of-fit on F^2^	1.068
Final R indices [I>2sigma(I)]	R1 = 0.0520, wR2 = 0.1482
R indices (all data)	R1 = 0.0706, wR2 = 0.1603
Largest diff. peak and hole (eÅ^−3^)	1.12→nd −1.08

**Table 2:** Cyclic Voltammetry Values of Various Complexes.

Entry	Compound	*E*_1/2_/V	ΔE/mV	*i*_c_/*i*_a_	Solvent
1	Ferrocene	0.460 ± 37	143 ± 26	0.97 ± 0.03	CH_2_Cl_2_
2	Keto-Ferrocenophane (1)	0.604 ± 20	115 ± 12	0.86 ± 0.04	CH_2_Cl_2_
3	Ferrocenophane (2)	0.318 ± 63	137 ± 21	0.87 ± 0.04	CH_2_Cl_2_
4	Ferrocene	0.400 ± 94	184 ± 40	0.98 ± 0.02	CH_3_CN
5	Keto-Ferrocenophane (1)	0.658 ± 70	327 ± 53	0.89 ± 0.06	CH_3_CN
6	Ferrocenophane (2)	0.395 ± 38	106 ± 10	0.94 ± 0.03	CH_3_CN

Conditions: glassy carbon working electrode, Pt counter electrode, Ag wire pseudoreference electrode. Solutions 1 mM in substrate, 0.1 M in *n*-Bu_4_NPF_6_ and flushed with argon. A scan rate of 100 mV s^–1^ was applied and the voltammograms were referenced to ferrocene (E° = 0.46 V in CH_2_Cl_2_, 0.40 V in CH_3_CN).

**Table 3:** Cyclic Voltammetry in presence of alcohol nucleophiles.

Entry	Compound	Nucleophile	*E*_1/2_/V	ΔE/mV	*i*_c_/*i*_a_	Solvent
	Ferrocene					
1		MeOH	0.324 ± 0.043	75 ± 30	0.94 ± 4	CH_3_CN
2		*n*-BuOH	0.301 ± 0.047	121 ± 20	0.78 ± 4	CH_3_CN
3		*i*-PrOH	0.339 ± 0.021	83 ± 5	0.96 ± 4	CH_3_CN
4		Propargylic alcohol **3**	0.410 ± 0.010	117 ± 31	0.88 ± 5	CH_3_CN
5		*n*-BuOH	0.404 ± 0.062	128 ± 26	0.94 ± 4	CH_2_Cl_2_
6		MeOH	0.352 ± 0.041	143 ± 19	0.94 ± 3	CH_2_Cl_2_
7		*i*-PrOH	0.302 ± 0.080	138 ± 8	0.95 ± 2	CH_2_Cl_2_
8		Propargylic alcohol **3**	0.344 ± 0.047	145 ± 12	0.94 ± 2	CH_2_Cl_2_
	Keto-Ferroceno-phane (**1**)					
9		MeOH	0.560 ± 0.032	76 ± 24	0.81 ± 0.04	CH_3_CN
10		*n*-BuOH	0.610 ± 0.050	73 ± 20	0.81 ± 0.04	CH_3_CN
11		*i*-PrOH	0.590 ± 0.040	72 ± 22	0.82 ± 0.05	CH_3_CN
12		Propargylic alcohol **3**				CH_3_CN
13		MeOH	0.553 ± 0.078	105 ± 7	0.84 ± 0.06	CH_2_Cl_2_
14		*n*-BuOH	0.616 ± 0.046	123 ± 23	0.84 ± 0.03	CH_2_Cl_2_
15		*i*-PrOH	0.432 ± 0.042	117 ± 22	0.87 ± 0.05	CH_2_Cl_2_
16		Propargylic alcohol **3**	0.648 ± 0.015	109 ± 12	0.75 ± 0.09	CH_2_Cl_2_
	Ferroceno-phane (**2**)					
17		MeOH	0.233 ± 0.022	75 ± 20	0.97 ± 0.03	CH_3_CN
18		*n*-BuOH	0.263 ± 0.048	96 ± 26	0.96 ± 0.04	CH_3_CN
19		MeOH	0.288 ± 0.010	108 ± 13	0.96 ± 0.04	CH_2_Cl_2_
20		*n*-BuOH	0.336 ± 0.007	137 ± 23	0.91 ± 0.02	CH_2_Cl_2_

Conditions: glassy carbon working electrode, Pt counter electrode, Ag wire pseudoreference electrode at a scan rate of 100 mV s^–1^. Solutions 1 mM in substrate, 0.1 M in *n*-Bu_4_NPF_6_ and flushed with argon in presence of the liquid nucleophiles (0.5 mL) or the propargylic alcohol **3** (0.004 g).

**Table 4:** *i*_c_/*i*_a_ values at different sweep rates in the presence of *n*-BuOH.

Entry	Sweep Rate mV/s	*i*_c/_*i*_a_ Ferrocene and *n*-BuOH		*i*_c/_*i*_a_ Ketoferroceno-phane 1 and *n*-BuOH		*i*_c/_*i*_a_ Ferroceno-phane 2 and *n*-BuOH	
		CH_3_CN	CH_2_Cl_2_	CH_3_CN	CH_2_Cl_2_	CH_3_CN	CH_2_Cl_2_
1	50	55 ± 4	92 ± 4	90 ± 2	83 ± 2	92 ± 6	95 ± 2
2	100	71 ± 5	95 ± 2	92 ± 4	83 ± 3	96 ± 2	91 ± 2
3	200	66 ± 6	92 ± 2	90 ± 2	79 ± 5	92 ± 6	88 ± 1
4	300	71 ± 2	90 ± 2	91 ± 4	77 ± 6	95 ± 4	87 ± 2
5	400	77 ± 4	90 ± 3	91 ± 6	75 ± 5	96 ± 2	86 ± 4
6	500	78 ± 3	89 ± 3	91 ± 2	70 ± 6	96 ± 2	91 ± 2

Conditions: glassy carbon working electrode, Pt counter electrode, Ag wire pseudoreference electrode at a scan rate of 100 mV s^–1^. Solutions 1 mM in the substrate, 0.1 M in *n*-Bu_4_NPF_6_ and flushed with argon in presence of *n*-BuOH (0.5 mL).

**Table 5:** Catalysis results.

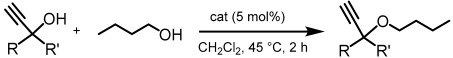				
Substrate	Product ^1^	Isolated Yield Fc^+^	Isolated Yield 1^+^	Isolated Yield 2^+^
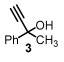	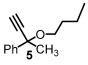	74% ^2^	0%	0%
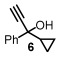	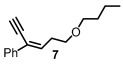	37% ^3^	13%	44%
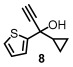	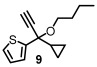	44% ^3^	-	6%

^1^ Reaction conditions: propargylic alcohol and alcohol in an equimolar amount, CH_2_Cl_2_ solvent at 45 °C for 2 h followed by chromatographic workup; ^2^ Data taken from [47]. ^3^ Data taken from [46].

## Data Availability

The CIF file of the finalized crystal structure **1**^+^SbF_6_ has been deposited in the Cambridge Structural Database and the reference ID is CCDC 2184543.

## Supplementary Materials

The following are available online at https://www.mdpi.com/article/10.3390/molecules28062729/s1, Synthetic procedures, Figure S1: Part of the crystal structure of **1**^+^SbF_6_, Figure S2: Molecular packing of the crystal, Table S1: Intermolecular interaction geometries of **1**^+^SbF_6_, plot of the *i*_max_ value vs the square root of the scan rates, IR and UV-vis spectra of **1**^+^SbF_6_ and **2**^+^SbF_6_, NMR spectra of catalysis products [95,96,97,98,99,100,101].
