# Supplementary material for: Ferrocenophanium Stability and Catalysis
Source: Molecules. 2023 Mar 17;28(6):2729. doi: 10.3390/molecules28062729 (PMC10058812; doi:10.3390/molecules28062729)
Supplement: Supplementary file 1 [file molecules-28-02729-s001.zip › molecules-2267775-supplementary.pdf]

Ferrocenophanium Stability and Catalysis

Sai Anvesh Bezawada, Neira Ušto, Chloe Wilke, Michael Barnes-Flaspoler, Rajamoni Jagan and Eike B. Bauer*

University of Missouri - St. Louis, Department of Chemistry and Biochemistry, One University Boulevard, St. Louis, MO 63121, USA; Tel.: +1 (314) 516-5311; Fax: +1 (314) 516-5342; bauere@umsl.edu.

Supplementary Information

Table of Content

General Section, ferrocenophanium syntheses S2

X-Ray structure determination Table S1 and
Figures S1 and S2 .... S3

Plots of i_{max} vs the square root of the scan rate S5

Synthesis of Catalysis Products in Table 2 .... S6

IR spectra of **1**$^{+}SbF_6$ (top) and **2**$^{+}SbF_6$ S8

UV-vis spectra of **1**$^{+}SbF_6$ (top) and **2**$^{+}SbF_6$ S9

NMR Spectra of catalysis products.............................. S10

References S12

General experimental procedures

Chemicals (n-Bu_4PF_6, ferrocene, phenyl methyl propargylic alcohol **3**, $AgSbF_6$, Celite®) were purchased from and Sigma Aldrich and were used without any further purification. Dry solvents were obtained by standard distillation procedures. Column chromatography was performed with silica gel 60 (70-230 mesh) or aluminAR® (Mallinckrodt). ^1H NMR spectra were recorded in $CDCl_3$ using a Bruker instrument (300 MHz) and referenced to a residual solvent signal.

Ketoferrocenophane **1** and Ferrocenophane **2** were synthesized according to the literature [1,2]. The propargylic alcohols **7** [3] and **9** [4] were synthesized according to the literature. The NMR spectra of the catalysis products matched those in the literature [5,6].

Synthesis of ketoferrocenophanium hexafluoroantimonate [7],$\mathbf{1}^+SbF_6$

Silver hexafluoroantimonate ($AgSbF_6$, 0.272 g, 0.00079 mol) was added to a solution of the ketoferrocenophane **1** (0.190 g, 0.00079 mol) in diethyl ether (15 mL). The reaction was run for 1 hour under nitrogen in the dark. The reaction mixture was then filtered through Celite® under a nitrogen atmosphere and the solvent is removed under vacuum to obtain the product $\mathbf{1}^+SbF_6$ as a dark blue solid (0.28 g, 0.00059 mol, 74%). IR (ATR, neat): $\tilde{v}$ = 3112 (m), 2962 (m), 1692 (m), 1618 (m) cm^{-1}; UV-vis (CH_2Cl_2) [8]: λ_{max} (ε) = 632 nm (554 M^{-1} cm^{-1}).

Synthesis of ferrocenophanium hexafluoroantimonate [9], $\mathbf{2}^+SbF_6$

The ferrocenophanium hexafluoroantimonate $\mathbf{2}^+SbF_6$ was obtained using the same procedure than for $\mathbf{1}^+SbF_6$ in virtual quantitative yield. IR (ATR, neat): $\tilde{v}$ = 3114 (m), 2904 (m), 1633 (m) cm^{-1}; UV-vis (CH_2Cl_2) [8]: λ_{max} (ε) = 623 nm (456 M^{-1} cm^{-1}).

Single crystal data collection

Good diffraction quality crystals of **1**$^{+}$SbF$_6$ were obtained directly from the reaction product, of which a suitable shapped crystal of size 0.08 × 0.05 × 0.02 mm was chosen for X-Ray diffraction expriment. Intensity data of the crystal was collected using Bruker D8 Venture Photon II Diffractometer equipped with micro-focus sealed tube (Cu-source) of wavelength 1.54178 Å. Correct unit-cell parameters were determined by collecting data from 60 frames in three different crystallographic zones followed by data collection with exposure time of 10s per frame and sacn width of 0.5˚. Data reduction, scaling and multi-scan absroption correction were carried out using SAINT-plus and SADABS in APEX3 software [10]. The crystal structure was solved by direct methods procedure using the SHELXS program [11] and refined by Full-matrix least squares procedure on F2 using the SHELXL-2018 program [12]. The intensity data was checked for missing symmetry elements and Twinng with the PLATON program [12] Hydrogen atoms were placed in geometrically idealised positions and constrained to ride on their parent atoms with C–H distances in the range 0.93-0.97(Å). Isotropic thermal parameters (U_{eq}) were fixed such that they were U_{iso}(H) = 1.5U_{equ}(C) for aromatic C–H group and U_{iso}(H) = 1.2U_{equ}(C) for methylene group respectively. The molecular graphics images of the molecules were drawn using OLEX2 [13] and Mercury 3.9 software [14]. The crystal structure refinement details are summarized in Table 1.

Table S1: Intermolecular interaction geometries of **1**$^{+}$SbF$_6$.

**D-H…A**	**D-H (Å)**	**H…A(Å)**	**D-H…A(˚)**	**Symmetry operation**
C10–H10…O1	0.93	2.41	3.215(10)	*x, 1/2-y, -1/2+y*
C12–H12A…O1	0.97	2.37	3.270(11)	*x, 1/2-y, -1/2+y*
C3–H3…F3	0.93	2.57	3.179(11)	*x, 1/2-y, -1/2+y*
C5–H5…F1	0.93	2.64	3.425(10)	*1+x, 1/2-y, 1/2+y*
C7–H7…F3	0.93	2.37	3.281(13)	*1-x, 1/2+y, 1/2-z*

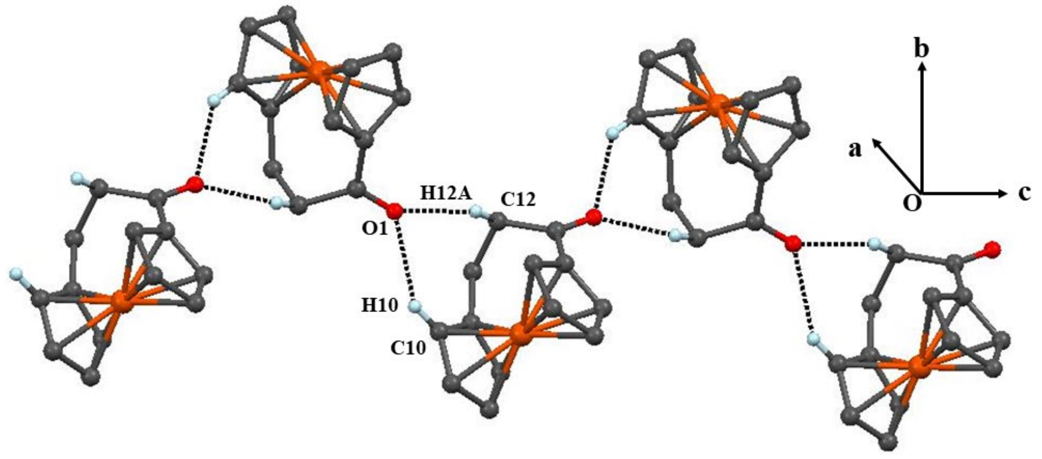

Figure S1: Part of the crystal structure of **1^{+}SbF$_6$** showing the formation of one-dimensional [3] ferrocenophan-1-one supramolecular chain via C–H…O interaction extending along the crystallographic [0 0 1] direction. The hydrogen atoms not involved in the hydrogen bonds are omitted for clarity.

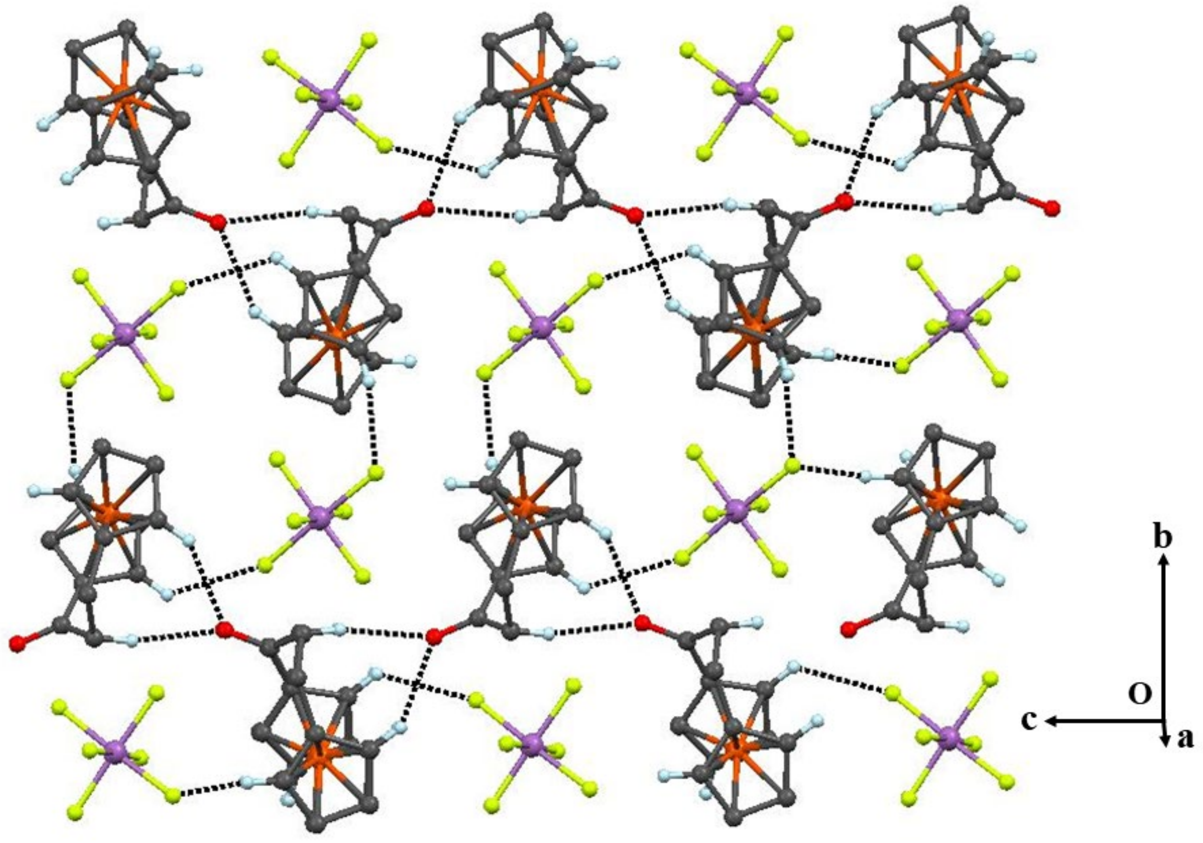

Figure S2: Molecular packing of the crystal viewed along the a-axis showing the formation of C–H…O and C–H…F interaction. The hydrogen atoms not involved in the hydrogen bonds are omitted for clarity.

Cyclic Voltammetry Traces – i_{max} vs $\sqrt{v}$ ($v = scan\ rate$)

A plot of the i_{max} value vs square root of the scan rate gives linear graphs, as expected for a diffusion-controlled electron transfer.

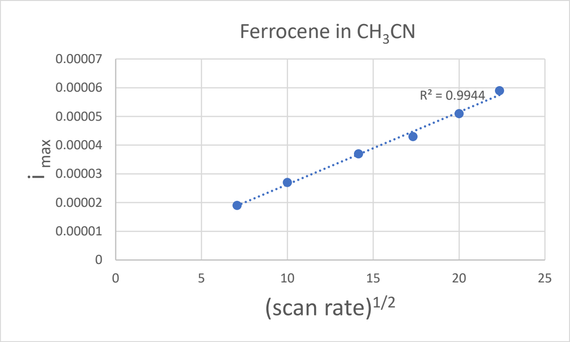

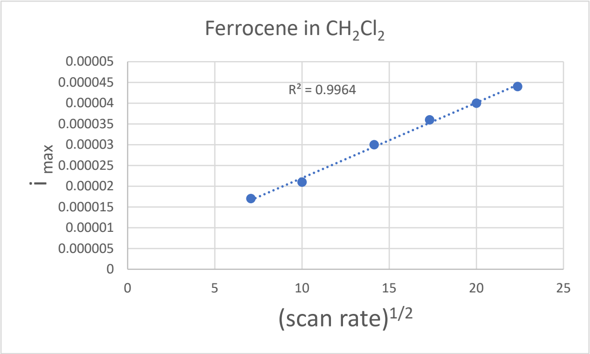

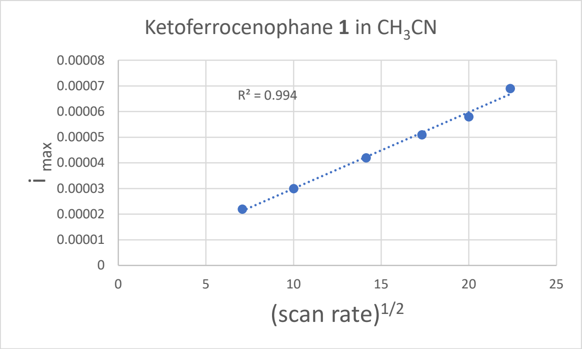

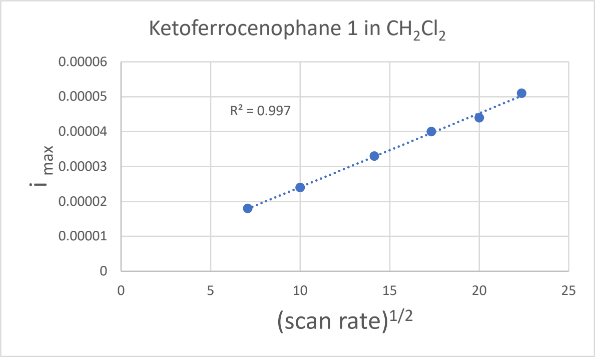

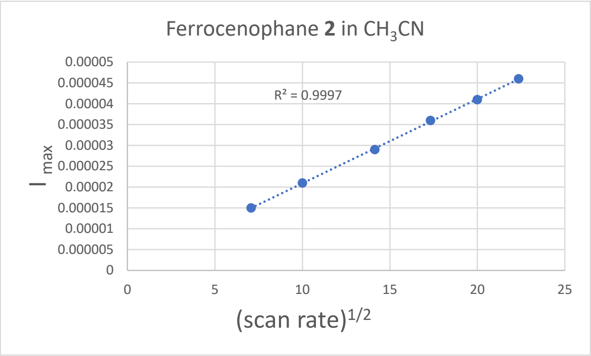

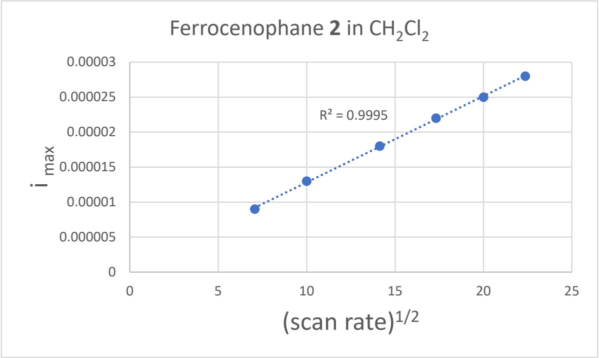

Experimental Section Catalysis (Table 5)

Phenyl-methyl-n-butyl ether (**5**) [6]: In a screw cap pressure vial, 1-phenyl-1-methyl-2-yne-1-ol (0.15 g, 1.06 mmol) was dissolved in CH_2Cl_2 (2 mL). *n*-butanol (0.075 g, 1 mmol) and the catalysts $\mathbf{1}^+SbF_6$ or $\mathbf{2}^+SbF_6$ (0.032 mmol) were added, and the vial was sealed and left at 45 °C for 4 h to 18 h. The reaction mixture was filtered through a short pad of silica gel with CH_2Cl_2 (2 mL) and the reaction was isolated by column chromatography using hexanes / ethyl acetate v:v 1:1; no appreciable amount of the product **5** could be isolated.

Ene-yne buty ether (**7**) [5]: In a screw cap pressure vial, 1-phenyl-1-cyclopropyl-2-yne-1-ol (**6**, 0.05 g, 0.29 mmol) was dissolved in CH_2Cl_2 (2 mL). *n*-Butanol (0.02 g, 0.29 mmol) and the ketoferroenophanium complex ($\mathbf{1}^+SbF_6$, 0.007 g, 0.015 mmol) were added, and the vial was sealed and heated at 45 °C for 2 hours. The reaction mixture was filtered through silica gel, using CH_2Cl_2 (2–4 mL). The product was obtained by column chromatography on alumina (2.5 × 30 cm, 9:1 v/v hexanes : ethyl acetate) as a yellow colored oil (0.009 g, 0.037 mmol, 13%). ^{1}H NMR (300 MHz, $CDCl_3$): δ =7.54–7.51 (m, 2H, aromatic), 7.29–7.18 (m, 3H, aromatic), 6.51–6.46 (t, 1H, J_{HH}=7 Hz, =CH), 3.52 (t, J_{HH}=7 Hz, 2H, OCH_2), 3.48 (t, 2H, J_{HH}=7 Hz, OCH_2), 3.27 (s, 1H, ≡CH), 2.79 (q, J_{HH}=7 Hz, 2H, CH_2), 1.55–1.45 (m, 2H, CH_2), 1.36–1.27 (m, 2H, CH_2), 0.85 (t, 3H, J_{HH}=7 Hz, CH_3) ppm.

Ene-yne butyl ether (**7**) [5]: 1-phenyl-1-cyclopropyl-2-yne-1-ol (**6**, 0.1 g, 0.58 mmol) was added to a 5-mL screw cap vial and dissolved in CH_2Cl_2 (1 mL). *n*-Butanol (0.043 g, 0.58 mmol) was added followed by the addition of the ferrocenophanium complex ($\mathbf{2}^+SbF_6$, 0.01 g, 0.03 mmol). The vial was then sealed and heated at 45 °C for 15 minutes. The sample was filtered through a short pad of silica and the solvent was removed. The reaction mixture was chromatographed on an alumina column (hexanes / ethyl acetate v:v 4:1) to obtain the product as a yellow oil (0.051 g, 0.254 mmol, 44%)

^{1}H NMR (300 MHz, $CDCl_3$): δ = 7.53–7.51 (m, 2H, aromatic), 7.28–7.18 (m, 3H, aromatic), 6.50–6.45 (t, 1H, J_{HH}=7 Hz, =CH), 3.57–3.52 (t, J_{HH}=7 Hz, 2H, OCH_2), 3.47–3.35 (t, 2H, J_{HH}=7 Hz, OCH_2), 3.28 (s, 1H, ≡CH), 2.72–2.67 (q, J_{HH}=7 Hz, 2H, CH_2), 1.51–1.44 (m, 2H, CH_2), 1.33–1.26 (m, 2H, CH_2), 0.87–0.82 (t, 3H, J_{HH}=7 Hz, CH_3) ppm.

Cyclopropyl thiophenyl butyl ether (**9**) [5]: In a screw-cap pressure vial, 1-thiophen-1-cyclopropyl-2-yne-1-ol (**8**, 0.050 g, 0.28 mmol) was dissolved in CH_2Cl_2 (1 mL) and *n*-butanol (0.020 g, 0.28 mmol) and the ferrocenophanium complex (**2**$^{+}SbF_6$, 0.005 g, 0.070 mmol) were added. Then the vial was heated at 45 °C overnight. The product was filtered through a short pad of silica gel using CH_2Cl_2 (2–4 mL). The product 1-thiophen-1-cyclopropyl-2-yne-1-ol (**9**) was obtained by column chromatography on alumina (2.5 × 30 cm, 9:1 v/v hexanes : EtOAc) as an orange colored oil (0.004 g, 0.016 mmol, 6%). ^{1}H NMR (300 MHz, CDCl3): δ =7.21–7.19 (m, 2H, aromatic), 7.13–7.12 (m, 1 H, aromatic) 6.89–6.87 (m, 1H, aromatic), 3.60 (dd, 1H, J_{HH} = 6 Hz, J_{HH} = 2 Hz, OC*H*H'), 3.25 (dd, 1H, J_{HH} = 6 Hz, J_{HH} = 2 Hz, OCH*H*'), 2.53 (s, 1H, ≡CH), 1.49–1.27 (m, 3H, CH+CH_2), 1.24–1.18 (m, 5H, CH), 0.83–0.78 (m, 6H, CH), 0.54–0.51 (m, 3H) ppm.

IR spectra of $1^{+}SbF_6$ (top) and $2^{+}SbF_6$

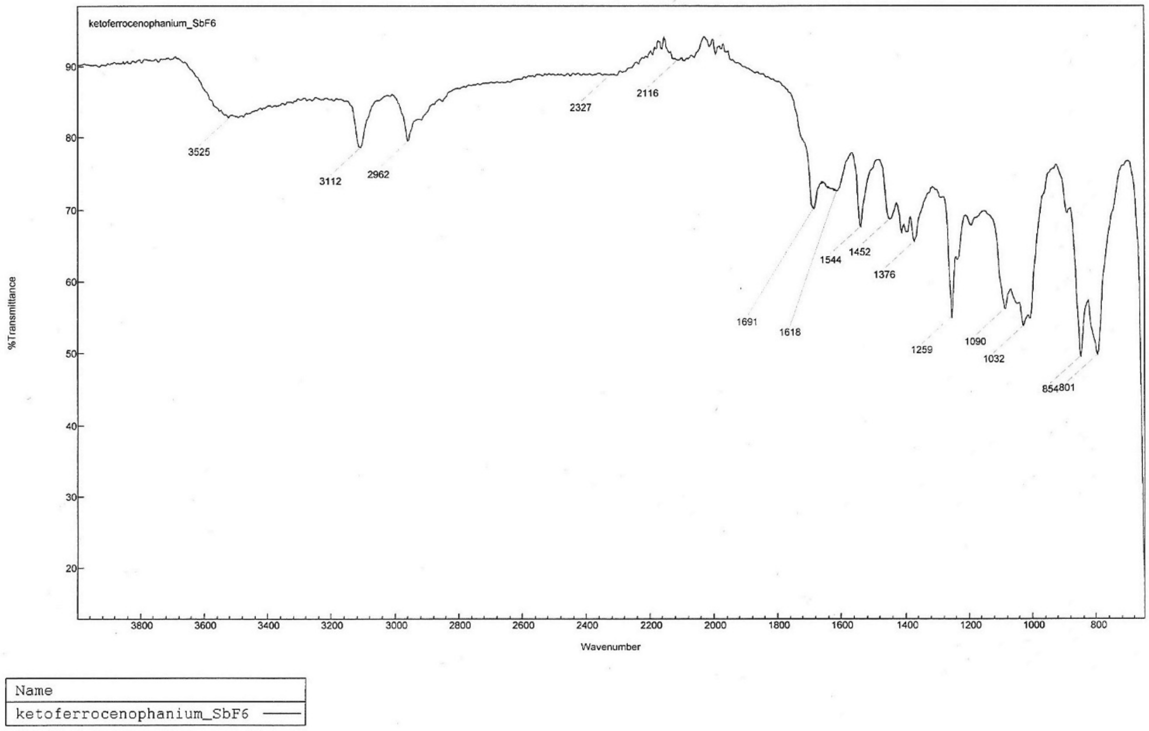

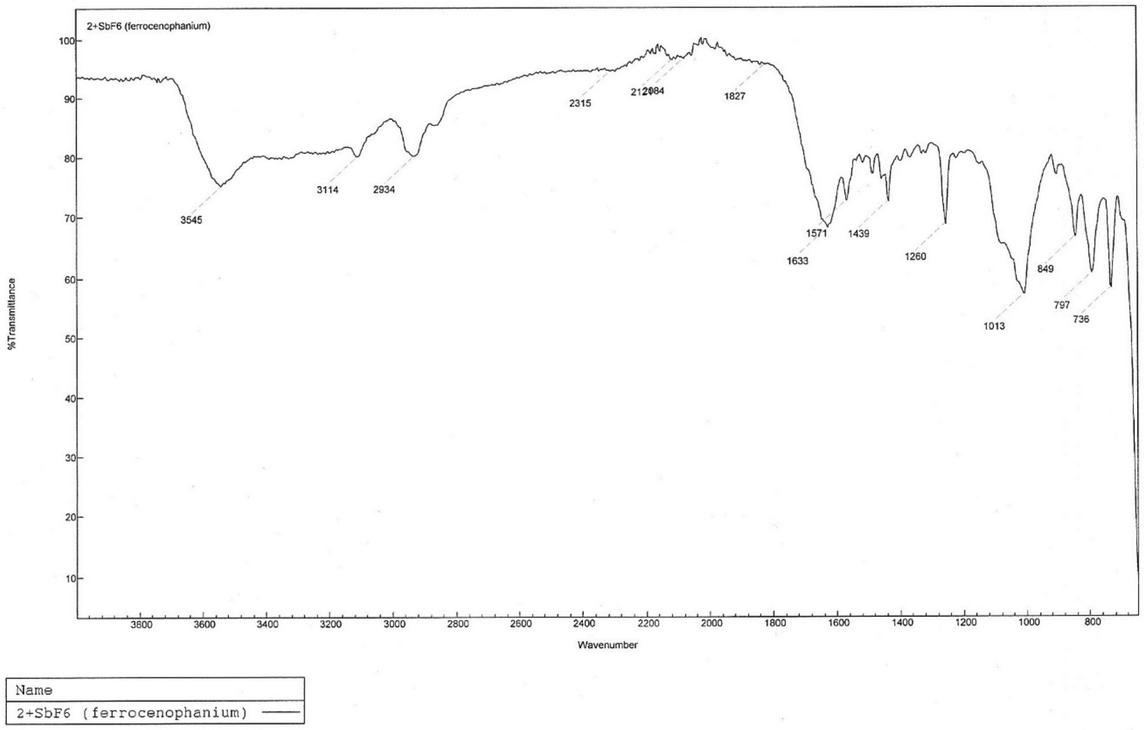

UV-vis spectra of $1^{+}SbF_6$ (top) and $2^{+}SbF_6$

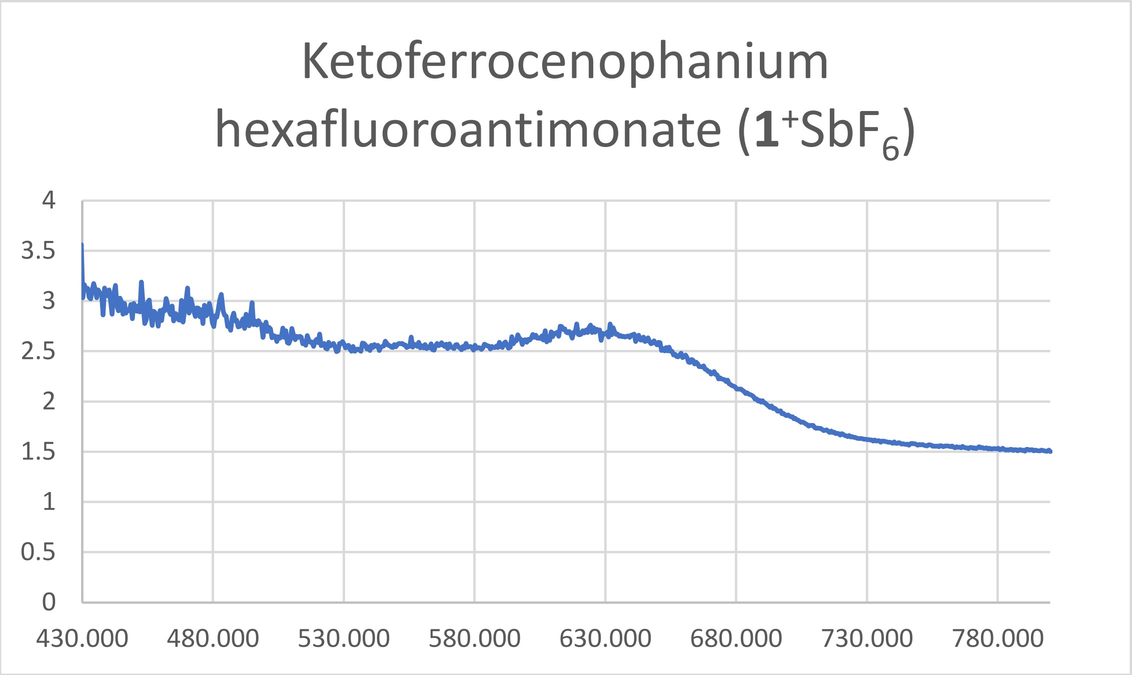

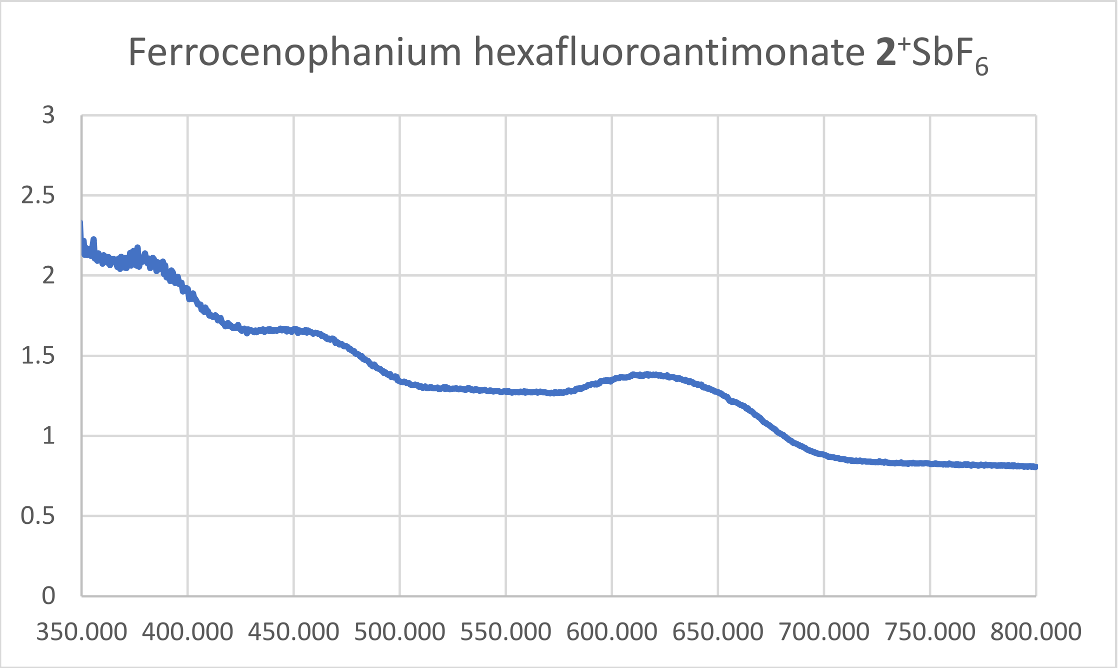

Table 5, compound 7. Employing the ketoferrocenophanium $\mathbf{1}^{+}$ (top) and ferrocenophanium $\mathbf{2}^{+}$ catalyst.

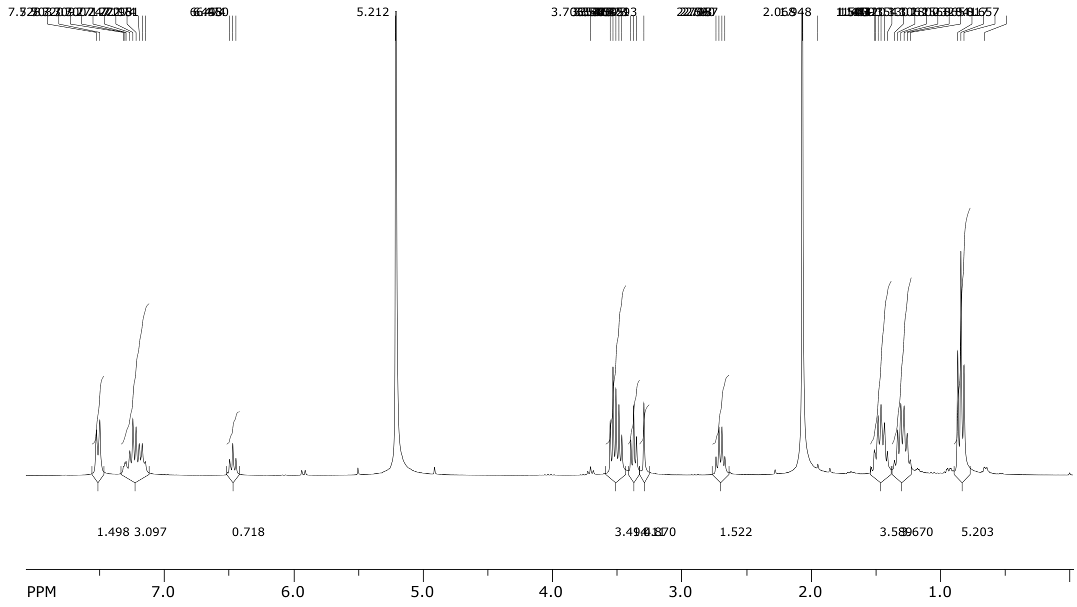

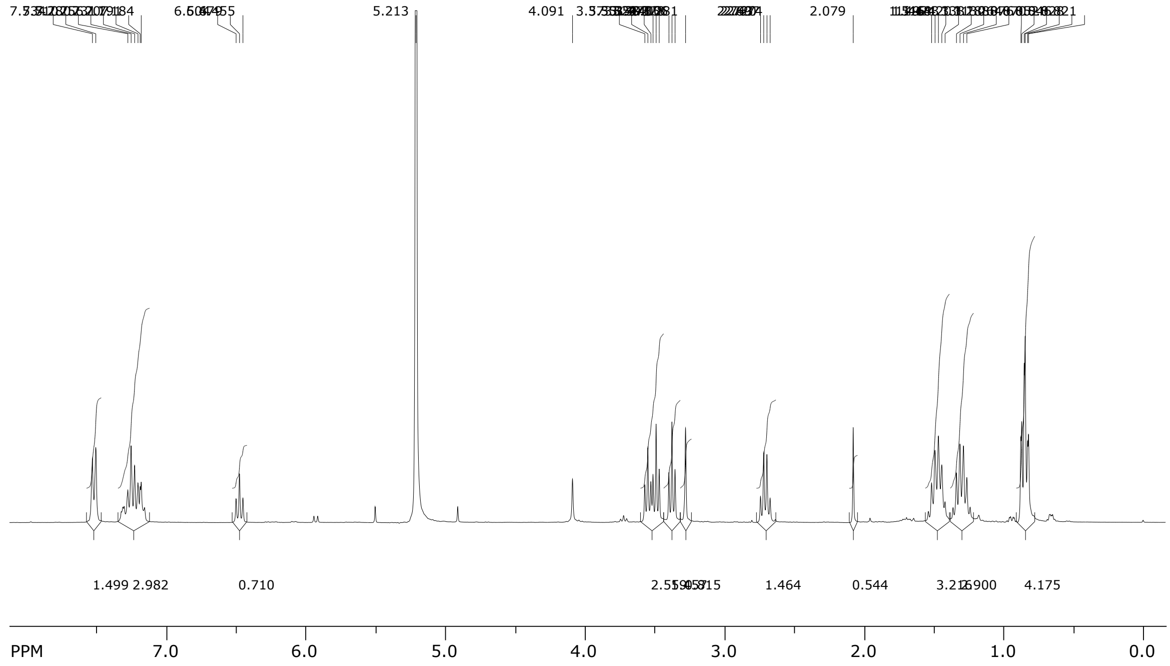

Table 5, compound 9. Employing the ferrocenophanium $\mathbf{2}^+$ catalyst.

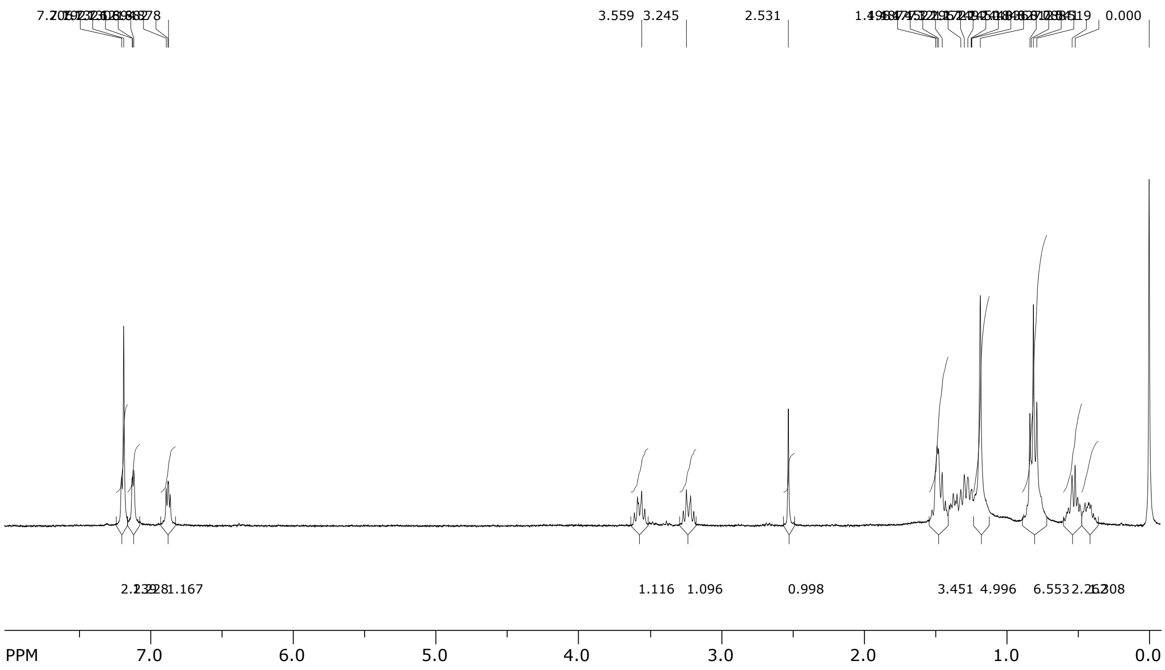

References

1 Turbitt, T. D.; Watts, W. E. Bridged ferrocenes: XII. The synthesis of [3] ferrocenophan-1-one from ferrocene by a novel one-step annelation reaction. *J. Organomet. Chem*. **1972**, *46*, 109–117.

2 Dong, T.-Y.; Lee, S. H. The effects of an interannular bridge on the electronic structure of ferrocenium cations *J. Organomet. Chem*. **1996**, *487*, 77–88.

3 Rao, W. Zhang, X.; Sze, E. M. L.; Chan, P. W. H. Ytterbium(III) Triflate-Catalyzed Amination of 1-Cyclopropylprop-2-yn-1-ols as an Expedient Route to Conjugated Enynes. *J. Org. Chem*. **2009**, *74*, 1740–1743.

4 Yamauchi, Y.; Onodera, G.; Sakata, K.; Yuki, M.; Miyake, Y.; Uemura, S.; Nishibayashi, Y. Ruthenium-Catalyzed Reactions of 1-Cyclopropyl-2-propyn-1-ols with Anilines and Water via Allenylidene Intermediates: Selective Preparation of Tri- and Tetrasubstituted Conjugated Enynes. *J. Am. Chem. Soc*. **2007**, *129*, 5175–5179.

5 Talasila, D. S.; Queensen, M. J.; Barnes-Flaspoler, M.; Jurkowski, K.; Stephenson, E.; Rabus, J. M.; Bauer, E. B. Ferrocenium Cations as Catalysts for the Etherification of Cyclopropyl-Substituted Propargylic Alcohols: Ene-yne Formation and Mechanistic Insights. *Eur. J. Org. Chem*. **2019**, 7348–7358.

6 Queensen, M. Q.; Rabus, J. M.; Bauer, E. B. Ferrocenium hexafluorophosphate as an inexpensive, mild catalyst for the etherification of propargylic alcohols. *J. Mol. Cat. A: Chem* **2015**, *407*, 221–229.

7 Carty, P.; Dove, M. F. A. The reaction of some ferrocenyl ketones with anhydrous silver tetrafluoroborate, a new route to substituted ferricenium salts. *J. Organomet. Chem*. **1971**, *28*, 125–132.

8 Watanabe, M.; Sato, K.; Motoyama, I.; Sano, H. Mössbauer Studies of Bridged Ferrocenophane Derivative's Polyiodides. *Chemistry Letters* **1983**, 1775–1778.

9 Duggani, D.M.; Hendrickson, D.N. Electronic structure of various ferricenium systems as inferred from Raman, infrared, low temperature electronic absorption, and electron paramagnetic resonance measurements. *Inorg. Chem*. **1975**, *14*, 955–970.

10 Bruker, APEX3, SAINT-Plus and SADABS (2016). Bruker AXS Inc., Madison, Wisconsin, USA.

11 Sheldrick, G. M. Crystal structure refinement with SHELXL. *Acta. Cryst*. **2015**, *C71*, 3–8.

12 Spek, A. L. checkCIF validation ALERTS: what they mean and how to respond. *Acta Cryst*. **2020**, *E76*, 1–11.

13 Dolomanov, O.V.; Bourhis, L.J.; Gildea, R.J.; Howard, J.A.K.; Puschmann, H. OLEX2: a complete structure solution, refinement and analysis program. *J. Appl. Cryst*. **2009**, *42*, 339–341.

14 Macrae, C. F.; Bruno, I. J.; Chisholm, J. A.; Edgington, P. R.; McCabe, P.; Pidcock, E.; Rodriguez-Monge, L.; Taylor, R.; van de Streek, J.; Wood, P. A. Mercury 4.0: from visualization to analysis, design and prediction. *J. Appl. Crystallogr*. **2009**, *41*, 466–470.
